# Supplementary material for: The Role of Resilience and Basic Hope in the Adherence to Dietary Recommendations in the Polish Population during the COVID-19 Pandemic
Source: Nutrients. 2021 Jun 19;13(6):2108. doi: 10.3390/nu13062108 (PMC8234050; doi:10.3390/nu13062108)
Supplement: Supplementary file 1 [file nutrients-13-02108-s001.zip › nutrients-1248812-supplementary.pdf]

Food frequency consumption

How often do you consume these types of foods.

While answering to the questions, please consider foods eaten currently during your meal times, between the meals as well as those eaten at home and when you are out.

In this part please give only one answer to each question.

		Frequency					
		a few times a day	once a day	a few times a week	once a week	1-3 times a month	Never
1	Vegetables						
2	Fruits						
3	Refined grains						
4	Whole grains						
5	Dairy products (no added sugar)						
6	Processed cheese						
7	White meat						
8	Red and processed meat						
9	Fish						
10	Legumes						
11	Butter/lard						
12	Oils/margarines						
13	Unsalted nuts, seeds						
14	Salty snacks						
15	Sweets						
16	Fast food						
17	Sweetened drinks						
18	Water						

1. Gender: male/female
2. Age (years) ……
3. Education
   a) Primary/vocational
   b) Secondary
   c) Tertiary
4. Place of residence
   a) Countryside
   b) Small town
   c) Town
   d) Big city

MEDGLISH
Katarzyna Kurczak
ul. Warszawska 3/20
05-100 Nowy Dwór Mazowiecki
NIP 531-145-91-63, Reg. 016450943
tel. 530 957 497
e-mail: medglish.kk@gmail.com

Nowy Dwór Mazowiecki, June 7th, 2021

WARSZAWSKI UNIWERSYTET MEDYCZNY
ul. Żwirki i Wigury 61
02-091 Warszawa

To Whom it May Concern,

it is hereby confirmed that the article entitled **'The role of resilience and basic hope in the adherence to dietary recommendations in the Polish population during the Covid-19 pandemic'** has been linguistically verified by Katarzyna Kurczak, MA, PhD, who is a Polish-English translator specializing in written medical translation. Copies of documents confirming the qualifications in translation are available upon request.
